# Multidetector Computed Tomography Findings of Acute Abdominal Intussusception Due to Peutz–Jeghers Syndrome

**DOI:** 10.7759/cureus.16401

**Published:** 2021-07-15

**Authors:** Hong Duc Pham, Thai Hoa T Nguyen, Thi Quynh Tran, Van Giang Bui

**Affiliations:** 1 Radiology, Hanoi Medical University, Ha Noi, VNM; 2 Radiology, Saint Paul Hospital, Ha Noi, VNM; 3 Medical Oncology, Vietnam National Cancer Hospital, Ha Noi, VNM; 4 Radiology, Vietnam National Cancer Hospital, Ha Noi, VNM

**Keywords:** peutz-jeghers syndrome, small bowel obstruction, intussusception, image findings, multidetector computed tomography

## Abstract

Peutz-Jeghers syndrome (PJS) is an autosomal dominant inheritance characterized by intestinal hamartomatous polyps and hyperpigmented mucocutaneous macules. Bleeding, bowel obstruction, and intussusception are the most common complications in PJS patients. Individuals are infrequently present for the first time with bowel obstruction secondary to intussusception. Intestinal intussusception presentation is often observed clearly on multidetector computed tomography (MDCT) with characteristic findings, such as “target” and “pseudo-kidney” signs, and sometimes shows the cause of lead-point polyp. A complemental examination is needed to attain more diagnostic symptoms of this disorder, including pigmented spots on the oral cavity and lips, family history with multiple gastrointestinal polyps. Here, we report a case of a 17-year-old male who showed traits of Peutz-Jeghers syndrome. However, the diagnosis was not made until he later developed bowel obstruction caused by an ileo-ileal intussusception manifestation on MDCT and eventually proved in typical hamartoma on postoperative histopathology.

## Introduction

Peutz-Jeghers syndrome (PJS) is a rare autosomal dominant disorder with the incidence estimated to be up to one in 200,000 live births, affected by a germline mutation in the serine/threonine kinase (STK11) gene on chromosome 19p13 [1]. PJS is characterized by multiple hamartomatous gastrointestinal polyps associated with mucocutaneous pigmentation of the lips, buccal mucosa, and digits [1,2]. Intussusception from PJS is one of the causes of acute intestinal obstruction with a clinical condition characterized by abdominal pain, requiring the clinician to make urgent treatment decisions. Various imaging techniques have been used to diagnose intussusception, including x-ray, ultrasound, and contrast enhancement studies. However, the use of multidetector computed tomography (MDCT) to diagnose the cause of abdominal pain has dramatically increased over the years. In addition, MDCT is helpful in providing valuable information about intussusception features [3]. We report a case of PJS in a 17-year-old male with complicated intussusception and its characteristic appearance on MDCT.

## Case presentation

**Figure 1:**
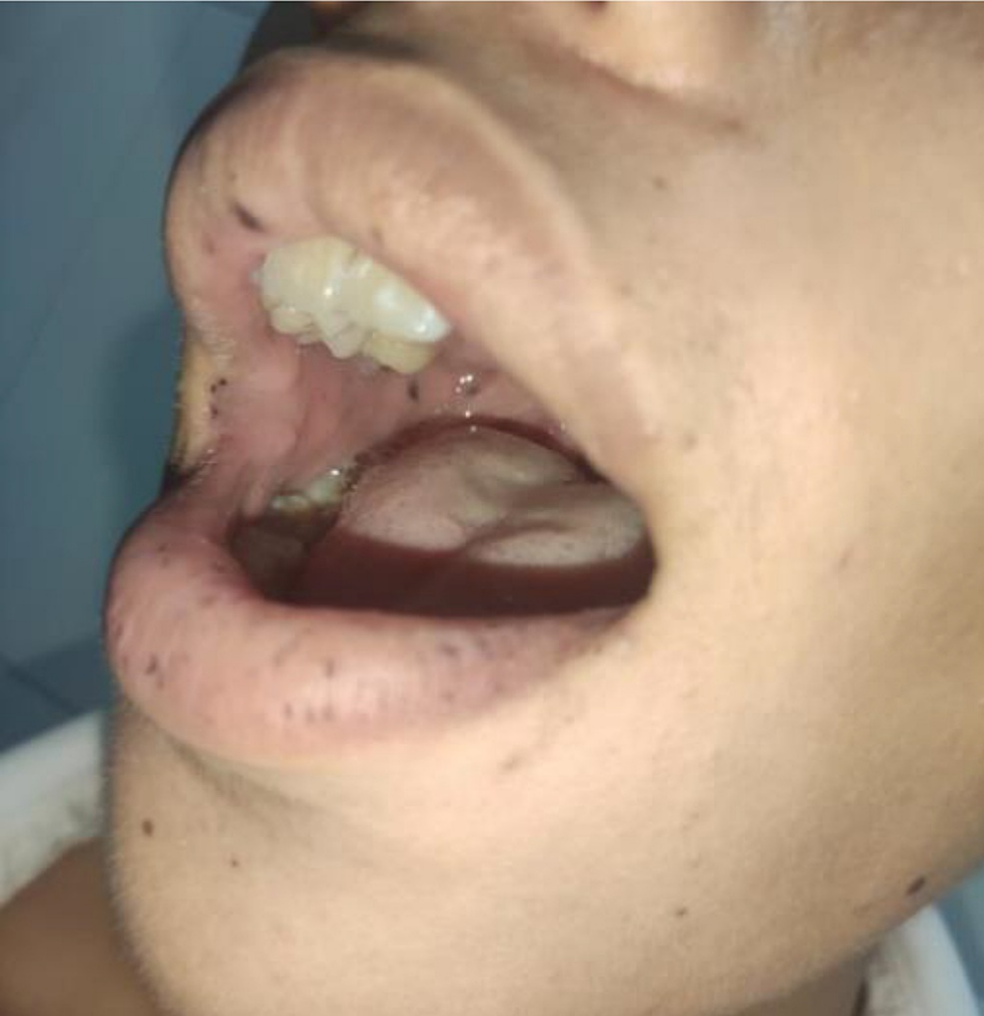
Photograph of the patient with the mouth open showing scattered melanin pigmentation spots on lips and buccal mucosa.

**Figure 2:**
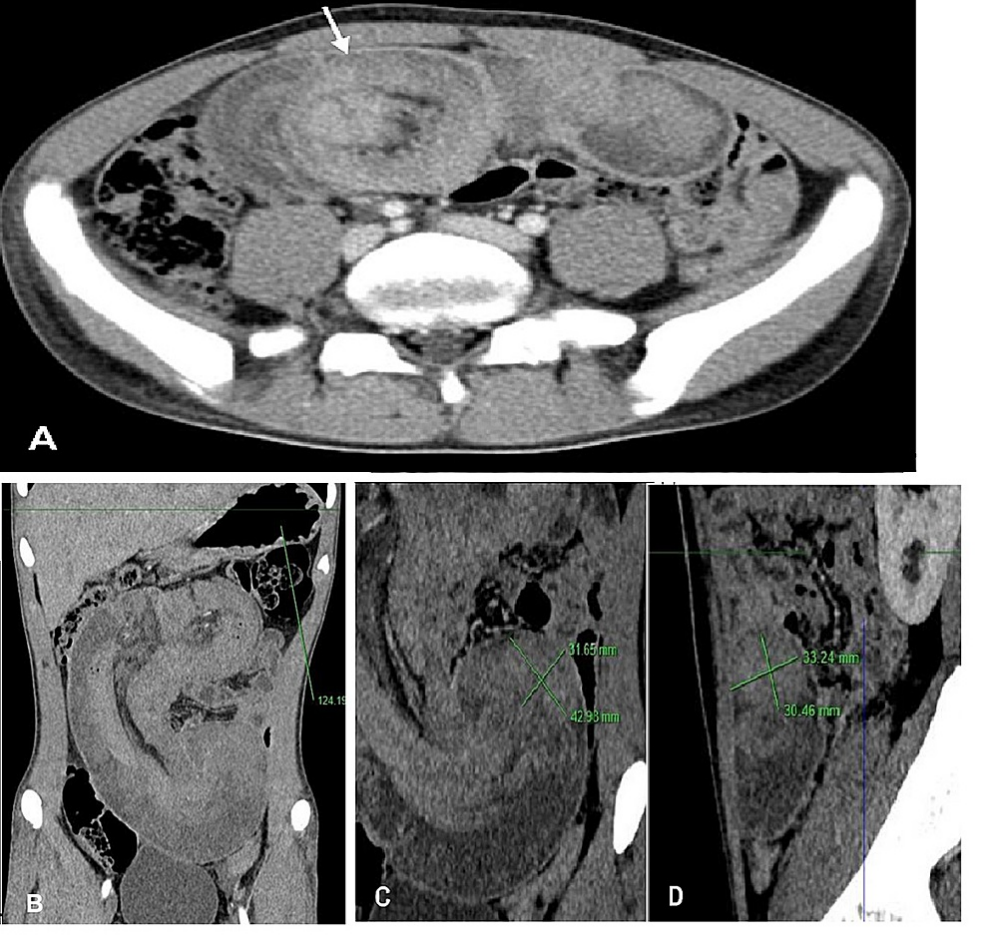
Enteroenteric intussusception presents a round mass as a “target” pattern on axial CT image (A) and as a large “sausage-shaped” pattern on coronal CT image (B), with a central hypodense area of mesenteric fat (arrow). Oblique (C) and sagittal (D) CT images depict a lead point of the mass at the apex of the jejunal intussusception.

A 17-year-old male was developed intermittent and slight episodes of upper abdominal pain over the preceding two months. He was presented to the emergency department with sharp abdominal pain and nausea without vomiting, which persisted for several hours. However, there was no history of surgery and other medical issues. One month ago, the patient performed the screening endoscopy for occasional abdominal pain and reported multiple tiny polyps in the stomach and colon. In addition, his father also has numerous polyps in the colon. Clinical examination revealed multiple pigmentations over the lips and buccal mucosa (Figure 1), but could not identify other lesions on the hands and feet, and a palpable abdominal mobile mass in mild abdominal distension. His vital signs were normal, with no fever and no bloody mucoid stools. Laboratory was unremarkable and so were ordinary tumor markers CA199 and CEA. Abdominal ultrasound revealed the large hyperechoic “target” and “pseudo-kidney” mass suggesting intussusception. MDCT was performed immediately after, which has clarified a small bowel intussusception with a lead point of the mass at the apex of the intussusception (Figure 2).

**Figure 3:**
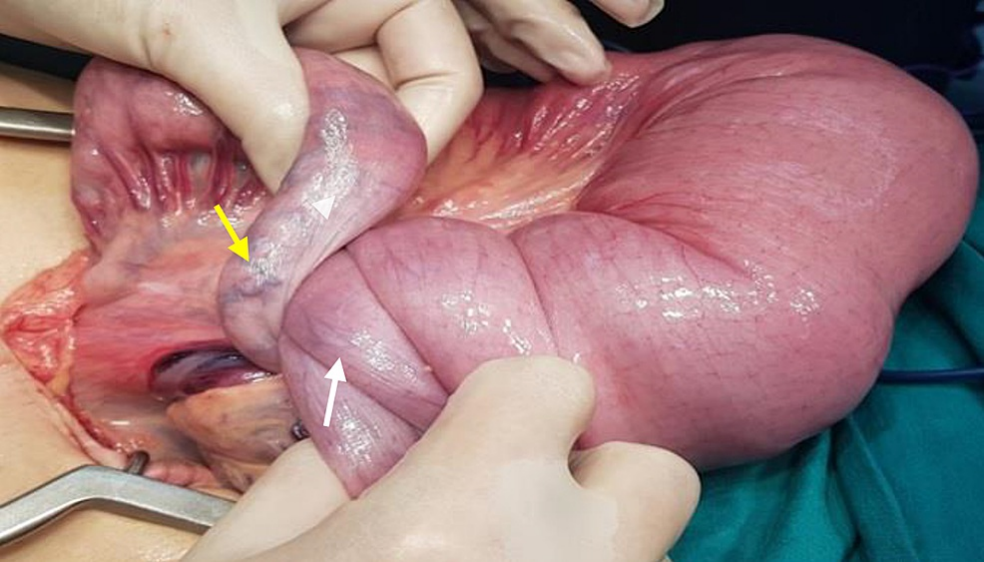
Intraoperative photograph demonstrating the transition zone of the intussusception. The intussusceptum (white arrow) is dilating and obstructing the intussuscipiens (yellow arrow).

The patient underwent exploratory laparotomy and confirmed a jejuno-jejunal intussusception of 30-cm long located 30 cm from the Treitz angle (Figure 3). The bottom of the intussusception was squeezed in the opposite direction of peristalsis to disinvaginate manually. It revealed that the cause of the intussusception was lead-point polyp (seen also on MDCT) measuring approximately 3.1 cm x 3.5 cm. About 8 cm above this polyp, the patient had a second polyp of the intussuscipiens measuring approximately 2.8 cm x 3 cm; roughly 30 cm below the lead-point polyp, the patient had a third polyp measuring around 2 cm x 1 cm. An investigation of the entire gastrointestinal tract was performed by endoscopy through enterotomy, and no other tumors were seen. The small bowel loops that were about 50-cm long carrying these three polyps with their mesentery were resected, and end-to-end enteric anastomosis was performed.

**Figure 4:**
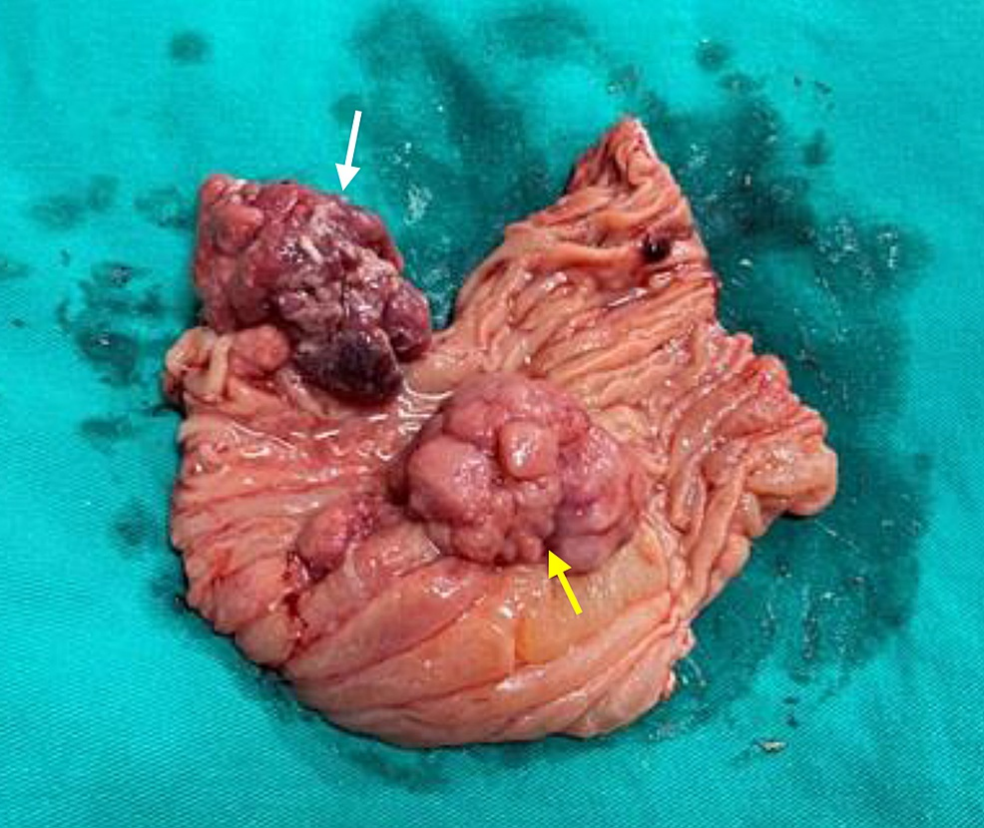
Photograph of the two sessile polyps of sizes 3.0 cm and 3.5 cm. The gross jejunal specimen shows the lead-point polyp about 3.5 cm in diameter in the edge of the intussusception; note the hemorrhagic necrosis over the surface of the polyp (white arrow). The yellow arrow shows another polyp with a size of 3.0 cm; the surface has cerebriform convolutions. There are also several smaller polyps aggregated at their bases.

**Figure 5:**
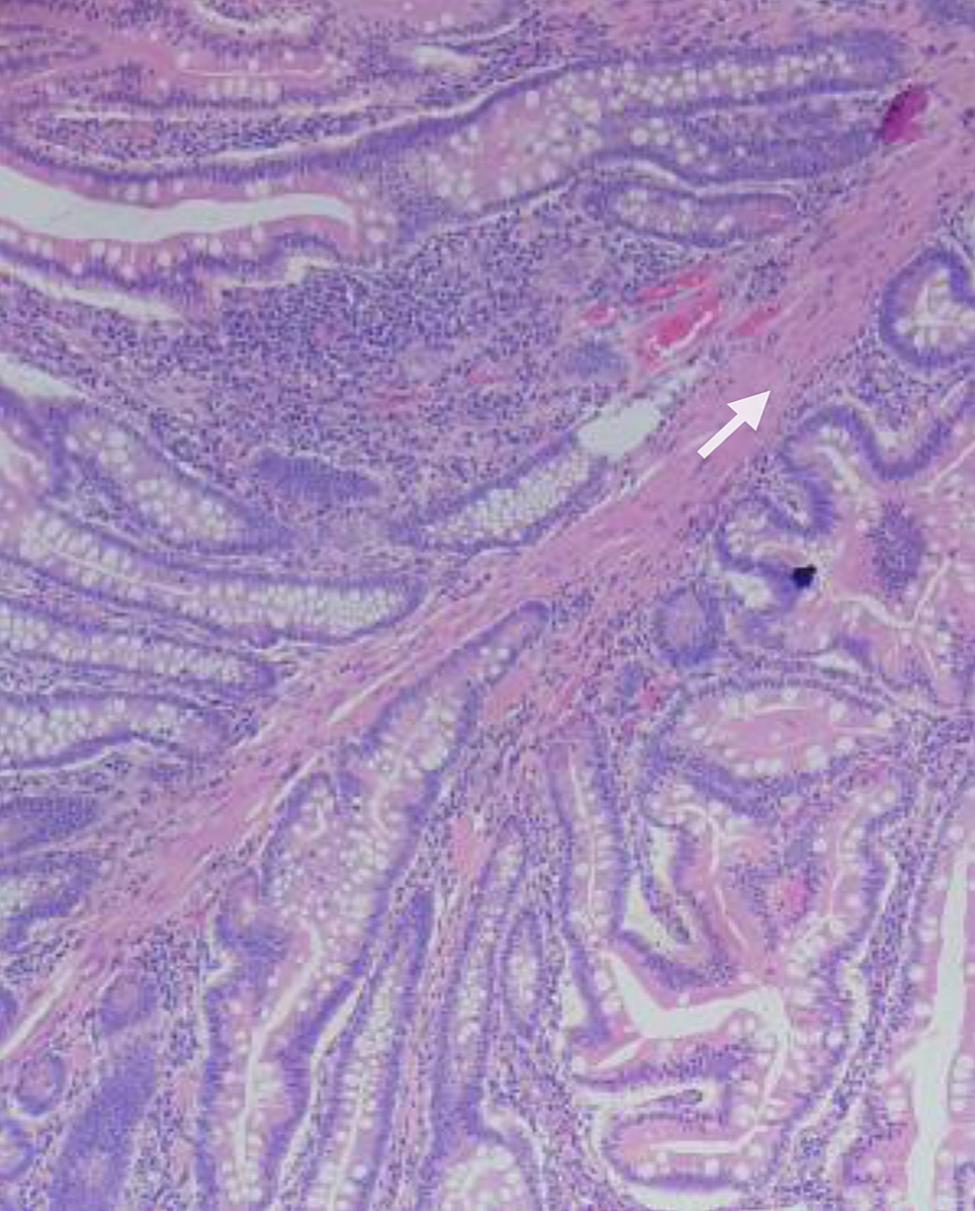
Photomicrograph (original magnification, x100; hematoxylin-eosin stain) demonstrates that a large polyp displays a tree-like structure of musculature (white arrow) covered with normal mucosa of the small bowel.

Macroscopically, the two sessile polyps causing intussusception were located next to each other with sizes of 3.0 cm and 3.5 cm; the surface showed cerebriform convolutions (Figure 4). Histopathology testing and diagnosis were done by two staining methods such as hematoxylin-eosin (HE) and periodic acid-Schiff (PAS). Specimens removed from the small intestine tissue have a tree-like structure with the axis of muscularis mucosa covered with normal mucosa cells that are round, small, and uniform. Some enlarged glands form cysts. Connective tissue increases fibrous proliferation and is rich in blood vessels; chronic inflammatory infiltrates without malignant cells were found (Figure 5). Thus, this histopathology confirms the clinical diagnosis of PJS.

After the operation, the boy was dismissed from the hospital in good condition; but he will still be monitored closely and regularly.

## Discussion

Polyps from PJS can occur anywhere in the gastrointestinal tract except the esophagus, and they are most commonly found in the small intestine in above 90% of patients, predominantly in the jejunum, followed by the ileum, and then duodenum [2]. Intussusception is a complication of PJS polyps and is the reason why the patient presented to the emergency department with acute abdominal pain, possibly with an anamnesis of one or more episodes of intussusception and may be reduced spontaneously, accounting for 69% of recurrent intussusception cases [4]. The age of the first intussusception due to PJS is variable; even from a young age, the reported youngest case was three years old [5]. The intussusception risk is 50% at the age of 20, with the most important risk factors being polyp located mainly in the small bowel in 95% of cases and a size of ≥1.5 cm in diameter for all ages. Other predictors, including sex, family history, and STK11 mutation status, were independent of developing intussusception risk [4].

Intussusception can pose a risk of bowel obstruction and even intestinal infarction, in which case, laparotomy is most commonly required to reduce the risk of these emergency complications. Before reaching 18 years of age, 68% of patients with PJS have undergone a laparotomy for an episode of intestinal obstruction [6]. Another study showed that up to 92.5% of intussusception cases were emergency surgery [4].

In a previous Dutch series, 80% of intussusception PJS presented as an acute abdomen regarding the definitive diagnosis. Among them, confirmation diagnosis was mainly reported intraoperatively and in several cases by medical history; only 8.5% of patients were diagnosed by imaging reports, including plain film, ultrasound, and CT scan [4]. The authors also reported a 33% mortality rate for intussusception. However, they did not discuss it, possibly because the lack of use of imaging modalities at the time delayed the assessment process preoperatively and may lead to an increased risk of complications such as intestinal necrosis.

The plain abdomen is known for its role in finding intestinal obstruction and pneumoperitoneum. Thus, it has little or no effect on the preoperative diagnosis of intussusception; the positive rate can be as low as 0% [7]. Unlike plain films, small bowel ultrasonography has a definite role in symptomatic PJS, which may be visualized intussusception, and tends to be more accurate than in lean children. The “target” or “doughnut” on the transverse view and “pseudo-kidney” signs on the longitudinal view are the classic features caused by the central hyperechoic mesenteric fat within the hypoechoic wall of the intussuscipiens [5,8]. Although ultrasound was used more frequently, it did not guarantee diagnosis on most occasions, such as bowel distension context or obesity patient or hands of experienced radiologists, and abdominal CT was recommended.

Because contrast-enhanced CT is increasingly common and is considered routine for determining the etiology of abdominal pain, it can easily detect the complicated intussusception in Peutz-Jeghers syndrome presenting clinically as intestinal obstruction. An intussusception appears as a complex soft-tissue mass composed of the inner intussusceptum and outer intussuscipiens creating a “bowel-within-bowel” configuration, separated by a crescent mesenteric fat attenuation into the intussusception, forming the classic three-layer appearance [8]. Enhancing vessels can be visualized within the mesenteric fat. Intussusception had two characteristic patterns depending on the direction of the x-ray beam: (1) a “bulls-eye” or “target-like” pattern when it is perpendicular to the longitudinal axis of the intussusception and (2) a “sausage-shaped” or “reniform” patterns when it is parallel to the longitudinal axis [3,7,9,10]. On MDCT images, the lead-point mass of polyp’s PJS may be identified at the apex of the enteroenteric intussusception with loss of the classic three layers. Because of the edematous hypovascular intestinal wall and lead mass, it is often difficult to distinguish between them (Figure 2) [11,12].

Entero-MRI can give images and diagnostic results similar to MDCT but are rarely indicated in emergency cases and young children; it is usually performed as part of its regular monitoring for the presence of polyps in the bowel [13] and in some cases of transient and/or asymptomatic intussusceptions [3].

About the management, recently Latchford et al. [14] published a review and recommended that a symptomatic child with intestinal intussusception from a PJS polyp should be urgently referred for surgical reduction, especially with a lead point such as a small bowel polyp. Radiological or endoscopic reduction is not applicable in these cases of intussusception. During laparotomy, patients should ideally undergo an intraoperative enteroscopy to clear the small bowel of other PJS polyps as well as our case.

## Conclusions

Contrast-enhanced CT is increasingly used to evaluate intestinal obstruction in abdominal pain and must be studied closely for signs of intussusception. Since the diagnosis is often not clinically suspected, the radiologist may be the first to suggest the diagnosis. In young patients with small bowel intussusception caused by lead-point polyps, PJS should be considered. Clinical helpful information to confirm the diagnosis includes scattered dark brown macules on the lips and buccal mucosa and/or family history. Early diagnosis and timely surgical treatment are essential to limit complications caused by intestinal infarction.
